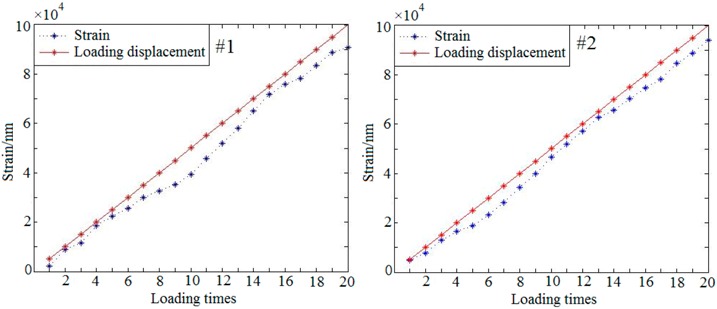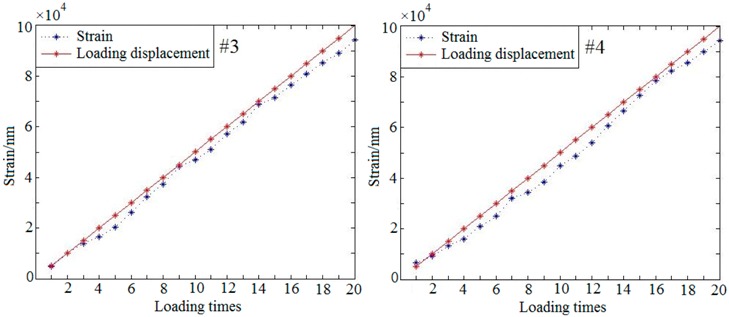# A Strain Distribution Sensing System for Bone-Implant Interfaces Based on Digital Speckle Pattern Interferometry

**DOI:** 10.3390/s19020365

**Published:** 2019-01-17

**Authors:** Ping Zhong, Zhisong Li, Huazheng Yang, Xin Tang, Guoxing He

**Affiliations:** 1 College of Information Science and Technology, Donghua University, Shanghai 201620, China; song272808216@163.com; 2 Department of Applied Physics, Donghua University, Shanghai 201620, China; shaohui_pan@163.com (H.Y.); geek_tx@163.com (X.T.); gxhe@dhu.edu.cn (G.H.)

**Keywords:** strain distribution detection, speckle interference imaging, phosphate buffer saline solution, digital speckle pattern interferometry, bone-implant interface

## Abstract

This paper aims to provide an effective measurement method for the distribution of deformations and strains focusing on the response to external loading of bone-implant interfaces. To achieve this target, a novel speckle interference imaging method is proposed by introducing phosphate buffer saline medium, in which the samples were completely placed into a phosphate buffer saline solution medium to stable the water molecules. The stability of interferometry imaging is analyzed by using the concepts of co-occurrence matrix and moment of inertia. A series of experiments to measure load-driven deformation and strain in the bone-implant interface was carried out, and the experiments results were analyzed and discussed. It shows that the proposed method is feasible and effective for the no-contact strain measurements of biomaterials in a physiological condition. The proposed strain distribution sensing system will contribute to evaluating computational simulations and improving selection of implant designs and materials.

## 1. Introduction

With the innovation of modern engineering and medical technology, multifunctional prosthesis have been widely used in the field of rehabilitation medicine [1]. However, there are still many problems that need to be solved in the installation of prosthetic devices. One major problem is how to fully understand the strain distribution. Determination of the distribution of strains and evaluation of potential stress mismatches in the interface are critically needed to prevent loosening, misalignment, potential infection in the interfacial gap, or induction of osteonecrosis [2]. Although computer-aided design (CAD) software and finite element modeling are useful for evaluation of deformation and strain [3,4,5,6], those models need to be validated by accurate measurements with proper boundary conditions and material properties. It is therefore important to experimentally measure load-driven deformation and strain in the bone-implant interface. Since bone is a non-homogeneous, anisotropic material, measurements of microscopic deformations and strains are necessary to understand the biomechanical in the bone-implant interface. Unfortunately, few measurement systems can provide excellent reliability, repeatability, resolution and accuracy [7,8].

Existing approaches include: (a) strain gauges or strain gauge arrays; (b) photogrammetry; and (c) modern optical measurement such as digital holographic (DH), laser speckle correlation (DSCM) and traditional digital speckle pattern interferometry (DSPI). Strain gauges and strain gauge arrays are relatively inexpensive, but they provide only localized information and the spatial resolution is severely restricted by the size and the shape of gauges [9,10]. Photogrammetry is a relatively robust technique, but this method has limited accuracy and is difficult to meet the requirements of resolution [11,12]; Optical measurement includes digital holographic (DH), digital laser speckle correlation (DSCM), conventional DSPI, etc. [13,14,15,16]. Among these measurement methods, DH has been heralded as a new era of whole-field deformation analyses in a microscopic level [16,17,18], however, DH is susceptible to changes in the external environment, such as vibration, and air disturbance [19,20]. The DSCM is suited for samples with a planar surface and not any curved surface, which will cause a significant error or measurement deviation [21,22]. DSPI, originally called as electronic speckle pattern interferometry (ESPI) [23,24], is considered as the most reliable technique for measuring microscopic deformation and strain [25,26,27,28,29,30,31], and many scholars have applied the DSPI technology to measure the strain in many fields [32,33,34].

In bio-material strain detection area, the DSPI technology has already achieved a wide application in dry bio-material, such as femurs, bird beaks, skulls, trabecular, cortical bones, mandibles, meniscus, teeth structures, etc. [35,36,37,38,39]. Nevertheless, this technology is used very limited and restricted in fresh bio-material. For example, Gaudette measured the surface deformations of epicardial heart based on the DSPI technology, in which he placed carbon-silicon particles on the surface to change the characteristics of the wet surface [40]. Kemper combined an endoscope with a DSPI device to detect the internal surface strain of pig stomachs [41], however, the result was noisy and fuzzy.

In this paper, a novel speckle interference strain distribution sensing system was proposed for measuring the distribution of deformations and strains focusing on the bone-implant interface in response to external loading. This paper is organized as follows: in Section 2, based on the stability analysis of DSPI imaging in the bone-implant interface, a new speckle interference imaging method was proposed, and the feasibility of this method was evaluated. In Section 3, in order to prove the validity of the new imaging method, a detecting system was built and a series of experiments were carried out. Section 4 included analyzes and discussions. Section 5 summarizes our conclusions.

## 2. Method and System Design

Unlike pure metallic samples in many other engineering applications, the applications in biological research involve relatively soft samples with a wet surface or biomechanical interfaces between an extremely hard metallic implant and softer bone. The current state-of-the-art DSPI systems cannot be directly applied to accurately determine strains for a wet or semi-wet surface.

### 2.1. Stability Analysis of Wet-Surface Biomaterials

Unlike the dry biomaterial, the random motion of water molecule and the change of external environment make water molecule distribution on the wet-surface constantly change, which will lead to serious noise in speckle interference images, and even to no image. Thus, current DSPI systems are difficult to apply directly to strain detection of biomaterials.

In biology, the concept of biological water was formally proposed by Nandi and Bagchi [42], and before long some researches proved that there was a biological water layer on the surface of active biological tissue [43]. With the changes of external environment, the movement of water molecules promotes molecular layer fluctuation on the biological tissue surface. During the process of strain detection, the original physiological balance is constantly being broken with environment change, and a large amount of biological water on the complex contour interface will fluctuates drastically. At the same time, with the process of dewetting, the ultra-thin biological water layer will produce a large amount of film rupture and shrinkage [44]. These changes will lead to serious speckle de-correlation and even impossible to get speckle interference fringes image, so it is necessary to propose a new method which can preserve the original physiological condition of bone-implant interface to improve speckle correlation.

### 2.2. An Innovative Imaging Method for DSPI Technology

In this paper, the test samples were fresh bone samples with a wet-surface. The movement of water molecule on wet-surface will lead to the digital speckle de-correlation. Therefore, the traditional DSPI technology cannot obtain a clear interference fringe image under mechanical loading.

In order to achieve the strain detection on the wet-surface in response to external loading, a novel speckle interference imaging method is proposed by introducing phosphate buffer saline (PBS) medium, in which the samples are completely immersed into the PBS solution. This method can avoid dewetting process by preserving the original physiological condition and realize the stability of liquid molecules on the macro probability. By using the method, it will reduce the molecular layer fluctuation on the detected surface, improve the speckle correlation and achieve the application of DSPI technology.

### 2.3. The Feasibility Analysis of Imaging Method

To evaluate the feasibility of the method above, the concepts of bio-speckles, time history of the speckle pattern (THSP) and moment of inertia are introduced.

Bio-speckles refer to the form of speckles of scattered light that appear on the imaging plane when a laser (or coherent light) is irradiated to the surface of a biomaterial [45]. A lot of experiments have already proved that the speckle stability is directly related to the biological activity, although the speckles constantly move, deform, disappear, and multiply [46,47]. According to this principle, speckle stability can be used to estimate the quality of speckle interferometry imaging under two conditions (air and PBS solution).

In order to evaluate speckle stability, a certain number of time-series speckle patterns (the value of speckle image row pixels is set as certain number in this paper) are recorded on the same detection area. Same rows are selected from the recorded image sequence as sample rows. With these selected rows, a new image is reconstructed, called THSP. The rows represent different points of speckle pattern, and columns represent the intensity changes of same location with time (In the THSP, the temporal axis is calculated according to the frame rate of the camera and the number of time-series speckle patterns).

Once the THSP is acquired, the moment of inertia method is applied [48]. Based on this method, the co-occurrence matrix (COM) is built by the following equation:

(1) $$COM=\left[N_{ij}\right],$$

where *i* and *j* are pixel gray values and *N* is the times (*i* followed by the *j* within all the columns).

The COM is reconstructed by calculating *N*, and the element located *i*-th row and *j*-th column is equal to *N* in the COM. According to this method, if the COM belongs to a series of static speckle patterns, it will only have non-zero elements on the diagonal line and zero elements on the other locations. On the contrary, if the COM is reconstructed by a series of dynamic speckle patterns, it will appear as a cloud-like white pattern near the diagonal line. The shape of the pattern is decided by the quantity of elements and the distance away from the diagonal line, which can reflect the speckle activity.

The moment of inertia is calculated by the co-occurrence matrix, which can evaluate sample activity quantitatively. The equation can be expressed as [49]:

(2) $$IM=\sum_{i,j}M_{ij}{\left(i-j\right)}^{2},$$

where *M_ij_* represents the probability of *N_ij_* occurrence, where *M_ij_* can be expressed as:

(3) $$M_{ij}=N_{ij}/\sum_{j}N_{ij},$$

The *IM* value is acquired from a series of time-series speckle patterns, and this value also can be used to indicate the stability of bio-speckles.

**Figure 1:**
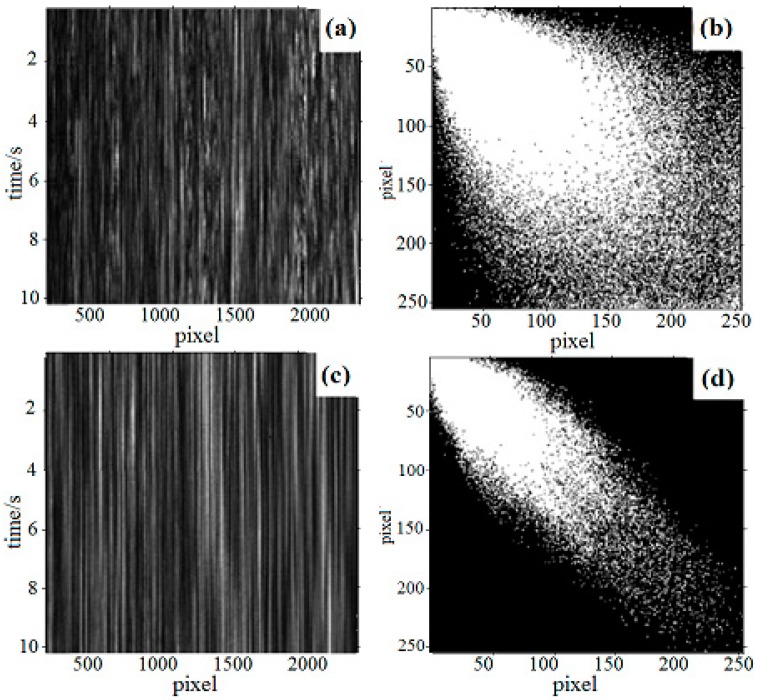
The comparison of time history of the speckle pattern (THSP) and Co-occurrence Matrix (COM). (**a**) THSP in air; (**b**) COM in air; (**c**) THSP in PBS solution; (**d**) COM in PBS solution.

For assessing the validity of this new speckle interference imaging method, an experiment was carried out using fresh bone samples. Based on the above principles and methods, the THSP images were reconstructed under two conditions (air and PBS solution), shown in Figure 1a,c, the corresponding COM of each condition is obtained as Figure 1b,d. From Equations (2) and (3), the *IM* values were calculated as 220.6 and 78.2, the result proved that the following relations are established:

(4) $$IM_{air}=220.6>IM_{PBS}=78.2,$$

The contrastive experiments show that the value of *IM* is smaller in PBS solution medium than that in the air environment for the DSPI speckle interferometry imaging. It proves that the proposed DSPI imaging method can effectively improve the stability of speckle interferometry.

## 3. Experiments

Three experiments were carried out to verify the actual effect of the new imaging method proposed in the paper.

### 3.1. Experimental Systems and Samples

#### 3.1.1. Design of Sensing System and Schematic Diagram

In order to apply DSPI to measure the load-driven deformation and strain in the bone-implant interface, a new DSPI sensing system was designed, in which the wet-surface sample is completely immersed into PBS solution.

**Figure 2:**
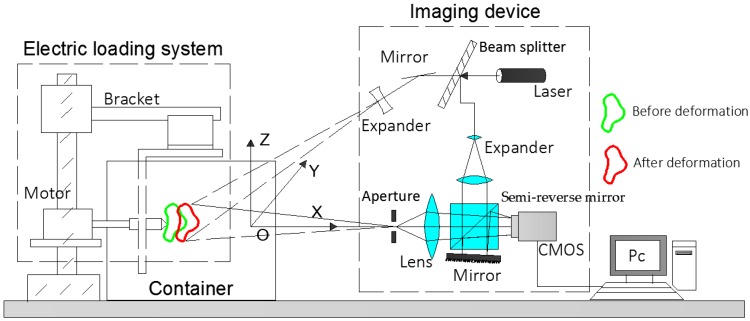
The schematic of the experimental system.

The schematic of DSPI sensing system is shown as Figure 2. The solid-state laser emitted a beam with a wavelength of 532.8 nm. According to the intensity, the coherent light source is split into a reference beam (5/100) and an object beam (95/100) by a beam splitter. The object beam is expanded by a beam expander (BE) and then illuminates the object. The diffused object light reflected from the object and goes through a slit aperture, imaging lens, semi-reverse mirror and arrives at the CMOS. On the other hand, the reference beam goes through another beam expander (BE), adjustable attenuator, semi-reverse mirror and reaches tilting mirror. After being reflected by the tilting mirror, the speckle pattern generated as a result of the interference between the reference beam and object beams is recorded by a CCD camera. The tilting mirror introduces a spatial carrier in the *x*-axis of the reference beam which allows the Fourier method to retrieve the optical phase [50,51]. The angle of the tilting mirror is related with the pixel size of the CMOS camera’s sensor and the illumination wavelength [52]. In order to avoid additional optical path difference in different transmission media, the front panel of this container was designed as a curved surface to match the imaging distance.

#### 3.1.2. Set-Up

**Figure 3:**
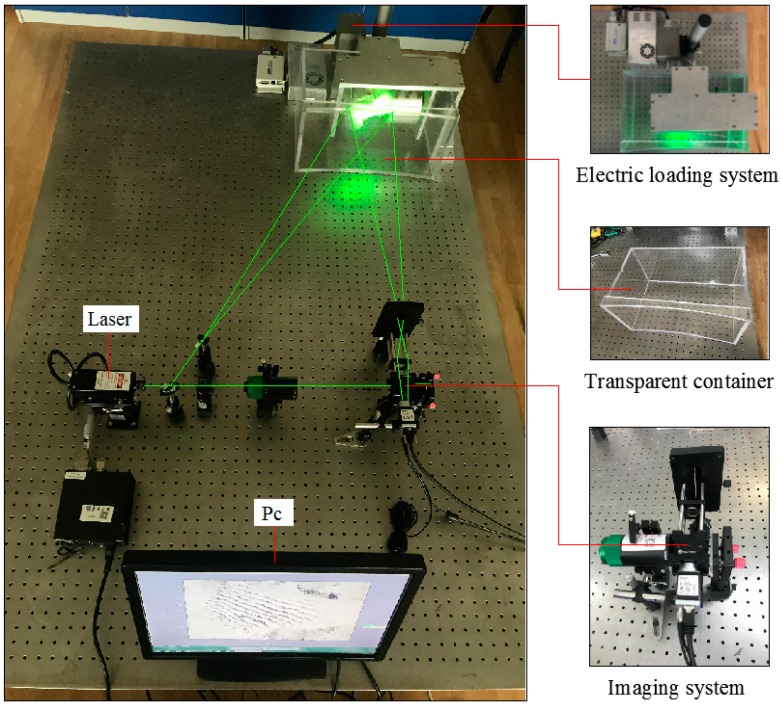
Experimental set-up for strain detection.

In order to achieve the measurement of load-driven deformation and strain in the bone-implant interface, an experimental set-up is shown in Figure 3. This set-up was composed of electric loading system, speckle interference imaging system, transparent container, PC, etc.

**Figure 4:**
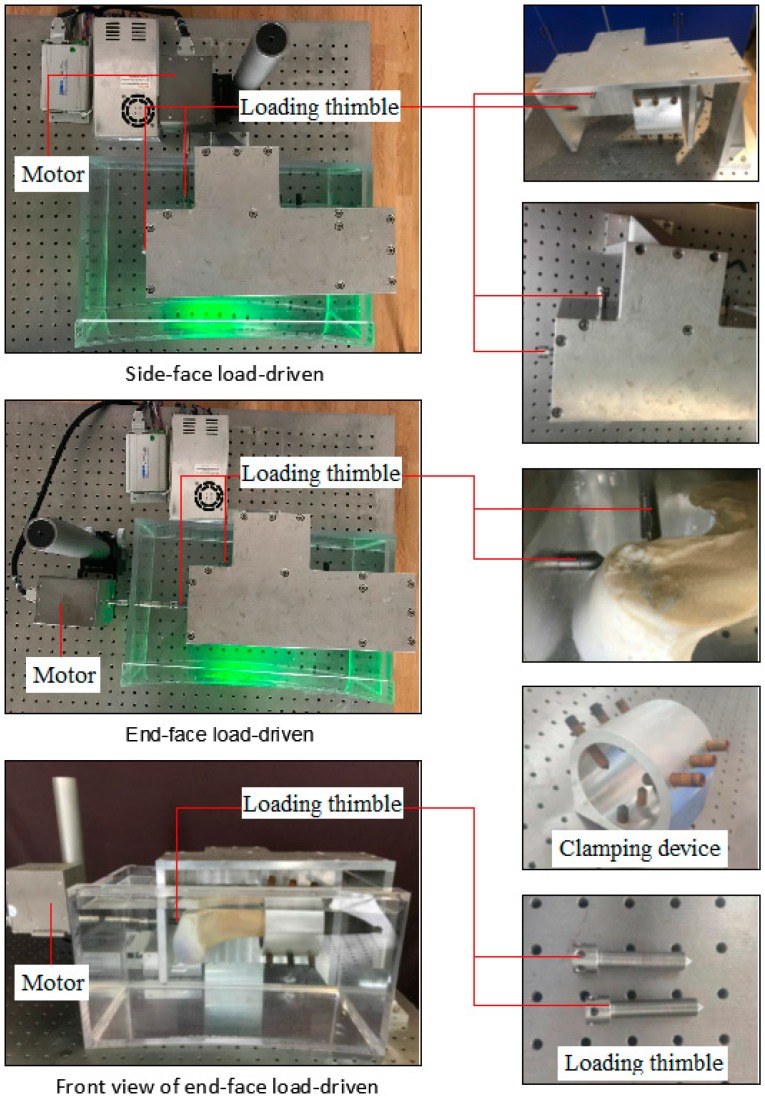
Physical diagram of sample installation.

The electric loading system includes a voice coil motor (linear resolution standard is 0.5 μm), control system, clamping device, loading thimble, etc. After a sample is installed and fixed by the clamping device, the clamping device together with the test sample will be placed into a transparent container which filled with PBS solution; then the voice coil motor will produce a certain displacement which will work on the sample to produce a strain. With the change of the loading points, the electric loading system can not only produce a radial but also a lateral displacement. Figure 4 showed the physical diagram of sample installation.

#### 3.1.3. Wet-Surface Biological Bone Samples

**Figure 5:**
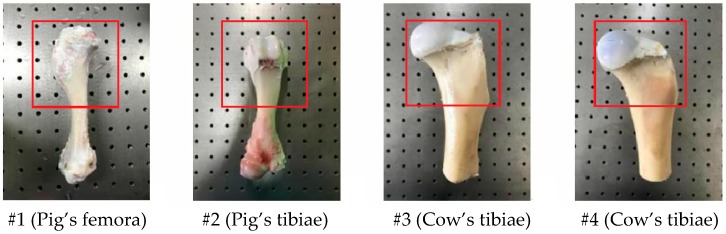
Wet-surface bone samples (Sample number: #1~#4).

Ex vivo femora and tibiae are chosen as samples in this experiment. These samples are taken from the living organisms (such as pig’s and cattle’s femora) no more than two hours to replace human joints. Before the strain detection, the soft tissue of the samples should be cleared away as much as possible to avoid the deviation. Four sets of bone samples (sample number: #1~#4) are shown in Figure 5 (The red rectangle area is the testing section).

### 3.2. Experiment Data Processing

#### 3.2.1. Speckle Image Preprocessing

In the DSPI technology, the noise will lead to a low peak signal-to-noise ratio (PSNR) and severely influences the subsequent accuracy [17,19]. During the speckle interference experiment, because of the random factors influence, a large amount of noise will be occurred. Considering the dynamic characteristics of the noise, a time-domain mean filter (TDMF) was used to eliminate the noise.

For obtaining excellent signal-to-noise ratio, a certain number of time-series speckle patterns (in this paper, this number is equal to 5) are recorded on the same area basing on the principle of TDMF [53]. According to averaging algorithm [54], every pixel on same position from these patterns is averaged to constitute a new filtered image.

**Figure 6:**
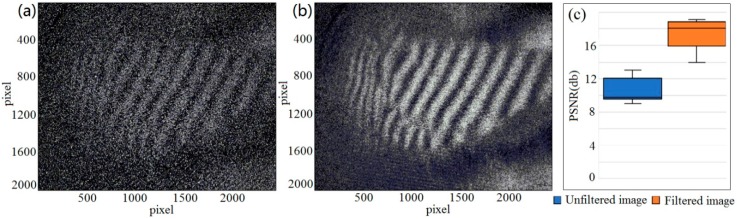
The comparison images of time-domain mean filter, (**a**) original image of interference fringe pattern, (**b**) filtered image by TDMF, (**c**) PSNR of original image of (**a**) and the filtered image of (**b**).

To evaluate the effect of the filter proposed in this paper, load-driven deformation and strain experiment is carried out and Figure 6a is the original image of interference fringe pattern which comes from the sample (#1) in response to external loading. The actual physical size of the test area approximately is 15 cm × 12 cm. Figure 6b shows the filtered image by TDMF and Figure 6c shows the peak signal to noise ratios (PSNR) come from the original image of Figure 6a and the filtered image of Figure 6b respectively. From Figure 6c, we can clearly see that the peak signal-to-noise ratio of the filtered image is significantly higher than that of the original image.

#### 3.2.2. Phase Extraction Technique and Calculation of Out-of-Plane Deformation

We know from the principle of digital speckle pattern interferometry that the strain distribution is directly related to the phase distribution, so how to extract the phase distribution effectively and accurately is crucial [55]. The spatial phase-shifting method can acquire the phase information from single image [56], which is suitable for dynamic or real-time strain measurement. Figure 3 shows the devices how to achieve a spatial phase-shift. It utilizes an adjustable mirror to carry the reference beam, which will bring a spatial frequency carrier. This reference beam is tilted a certain angle to illuminate on the CMOS; a spatial phase-shift will be created by this angle on the spatial frequency domain. Assuming wave fronts of object beam (*U*_1_) and reference beam (*U*_2_) can be written as:

(5) $$U_{1}\left(x,\;y\right)=\left|u_{1}\left(x,\;y\right)\right|\exp\left[i\varphi \left(x,\;y\right)\right],$$

(6) $$U_{2}\left(x,\;y\right)=\left|u_{2}\left(x,\;y\right)\right|\exp\left(-2\pi if_{0x}\cdot x-2\pi if_{0y}\cdot y\right),$$

where *f*_0*x*_ = (sin*θ*_0*x*_/*λ*), *f*_0*y*_ = (sin*θ*_0*y*_/*λ*), *θ*_0*x*_ is the angle between the reference light beam and the *x*-axis, *θ*_0*y*_ is the angle between the reference light beam and the *y*-axis and *λ* is the wavelength.

The recorded image can be expressed as:

(7) $$U(x,\;y)=(u_{1}+u_{2})\cdot (u_{1}*+u_{2}*),$$

By Fourier transformation:

(8) $$FT(U)=U_{1}^{2}+U_{2}^{2}+U_{1}(f_{x},f_{y})⊗U_{2}(f_{0x},f_{0y})*+U_{2}(f_{0x},f_{0y})⊗U_{1}(f_{x},f_{y})*,$$

Here ⊗ is the convolution operation. *FT*(*u*_1_) = *U*_1_(*f_x_,f_y_*), *FT*(*u*_2_) = *U*_2_(*f_x_,f_y_*). Among the four terms in Equation (8), the $U_{1}^{2}+U_{2}^{2}$ term is a low frequency term which mainly contains the background information and is located at the center of (*f_x_,f_y_*). The *U*_1_(*f_x_,f_y_*)⊗*U*_2_(*f*_0*x*_*,f*_0*y*_)* and *U*_2_(*f*_0*x*_*,f*_0*y*_)⊗*U*_1_(*f_x_,f_y_*)* terms are conjugated terms, which are located at (*f_x_*–*f*_0*x*_,*f_y_*–*f*_0*y*_) and (*f_0x_*–*f_x_*,*f_0y_*–*f_y_*), respectively.

An illuminating angle of the reference beam and slit aperture size are selected properly to separate the three spectra in the frequency domain. Then the phase can be obtained by the Windowed Fourier Inverse Transform (WIFT) and the complex amplitude calculations [57,58]. The phase before and after strain from load-driven can be expressed as:

(9) $$\left[j\left(x,y\right)+2pxf_{0x}+2pyf_{0y}\right]=\arctan\frac{Im[u_{2}u_{1}*]}{Re[u_{2}u_{1}*]},$$

(10) $$\left[j^{\prime }\left(x,y\right)+2pxf_{0x}^{\prime }+2pyf_{0y}^{\prime }\right]=\arctan\frac{Im[u_{2}^{\prime }u_{1}^{\prime }*]}{Re[u_{2}^{\prime }u_{1}^{\prime }*]},$$

**Figure 7:**
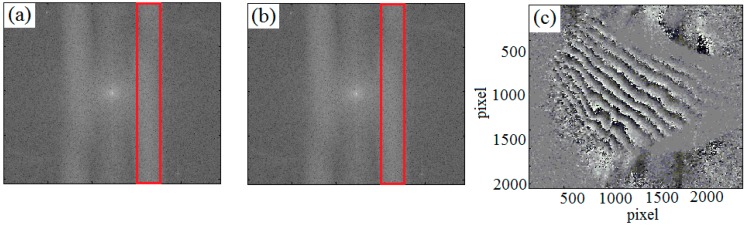
Spatial phase-shift testing results of out-of-plan strain, (**a**) frequency spectrogram of before deformation, (**b**) frequency spectrogram of after deformation, (**c**) phase difference diagram.

*Im* and *Re* are the imaginary and real part of the complex numbers. Figure 7a,b shows the frequency spectrograms of before and after deformation.

The phase difference (as shown in Figure 7c) can be calculated by the Equations (9) and (10):

(11) $$\Delta =\varphi (x,y)-\varphi^{\prime }(x,y),$$

The deformation can be obtained by Equation (12):

(12) $$\Delta =\varphi -\varphi^{\prime }=\frac{2\pi }{\lambda }(1+\cos\alpha )\tau ,$$

where, *α* is the certain illumination angle and *τ* is the out-of-plane deformation.

### 3.3. Experimental Results

In order to evaluate the effect of imaging method proposed in this paper, a series of experiments for the measurement of load-driven deformation and strain in the bone-implant interface were carried out.

#### 3.3.1. Contrast Experiment in Air and PBS Solution

**Figure 8:**
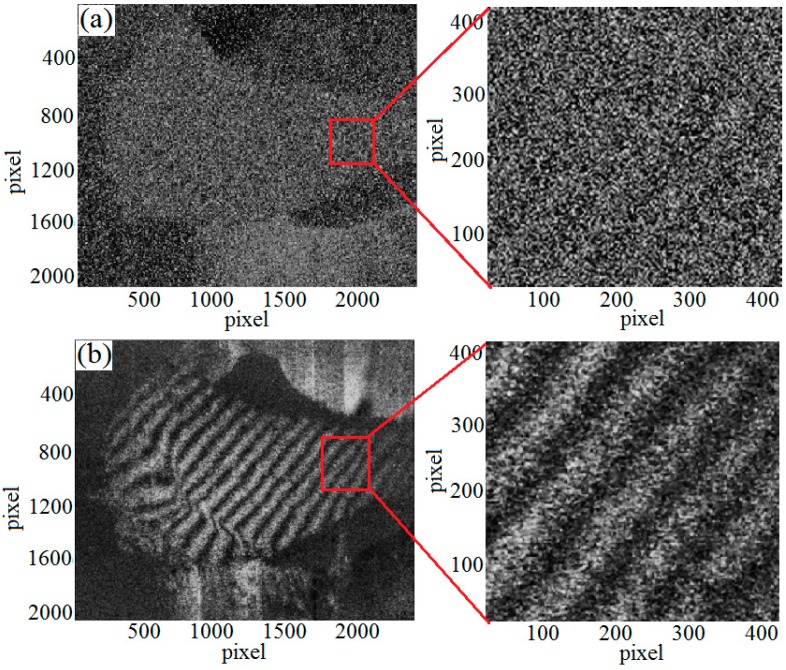
The interference fringe pattern (**a**) Speckle interference fringe pattern obtained in air (**b**) Speckle interference fringe pattern obtained in PBS solution.

Two experiments were conducted to evaluate the imaging effects under two conditions respectively. By using the mechanical loading device, an axial displacement was applied on the sample, and interference fringe patterns were measured, shown in Figure 8.

According to Figure 8a, it can be seen that the interference fringe pattern caused by load-driven deformation and strain is hardly obtained in the bone-implant interface in air. The reason is that the movement of water molecules promotes molecular layer fluctuation on the detection surface with the changes of biological tissue external environment in air, which will lead to speckle de-correlation, therefore, the DSPI technology cannot detect the deformation of fresh bone samples in an air environment. By contrast, Figure 8b shows that clear fringe pattern resulted from load-driven deformation and strain in the bone-implant interface can be obtained in PBS solution. When the bone samples that have wet surfaces or kept in a physiological aqueous environment are completely immersed into PBS solution, the original physiological environment of the bone sample is maintained to realize the stability of liquid molecules on the macro probability. The speckle correlation is improved, thus the correlative speckle patterns are recorded and clear fringe patterns can be obtained.

By comparing the fringe patterns, a conclusion can be drawn that the imaging result in PBS solution is better than in air environment, which proves that the interference imaging in liquid environment is an effective method for detecting the strain responded to external loading at the bone-implant interface.

#### 3.3.2. Multi-Sample Strain Detection Experiment

To further evaluate the effect and the scope of application of the new imaging method, a multi-sample strain detection experiment was performed. Four sets of samples were used to detect the strain which was caused by end-face load-driven. In order to observe the deformation, a phase unwrapping technique is required. The relationship between wrapping phase and unwrapping phase is shown as Equation (13):

(13) ϕ(x,y)=φ(x,y)+2kπ,

where ϕ(x,y) is the unwrapping phase information, φ(x,y) is the wrapping phase information, and *k* is a positive integer.

According to the Equations (12) and (13) and the least-squares method [59], the strain diagrams could be calculated. Based on the selected sample (#1~#4), the phase diagrams and strain diagrams which caused by end-face load-driven were obtained respectively. The experimental data of multi-sample in response to external dynamic loading are shown in Table 1.

#### 3.3.3. Quantitative Loading Experiment

**Figure 9:**
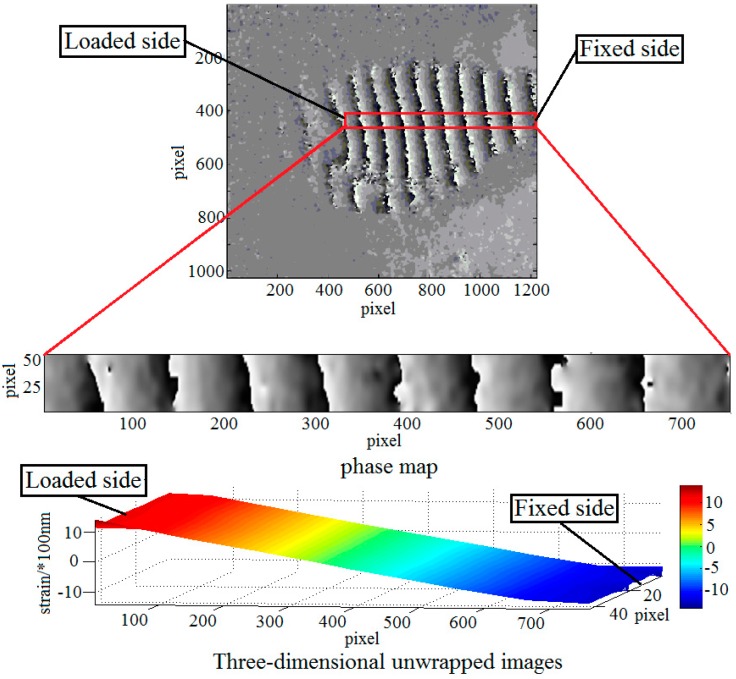
The quantitative loading experiment.

In order to evaluate the accuracy of the proposed method, a quantitative loading experiment was performed. The strain of the sample is caused by loading on the bone side-face. With this loading method, the out-of-plane deformation responded to external loading is unidirectional, which will be advantageous to calculate the deformation. The result of the strain was shown in Figure 9. The red rectangular area is the selected strain observation area on the phase map. In this area, the left end is the loading point, and the right end is the rigid fixed end. In theory, the strain of the red rectangular should be consistent with the magnitude of loads. According to the difference between the strains and the loads, train detection accuracy can be analyzed.

Based on this method above, a repeated test was carried out for the strain detection. In order to obtain the relationship between the strains and the loads, a periodic load (the displacement load per step is 5 μm) was applied to the samples. Each sample (#1~#4) was loaded 20 times. As the result shown in the Table 2, the *x*-axis represents the loading times and the *y*-axis represents the strain. The red solid line represents the loading displacement and the blue dotted line represents the detected strain.

The correlation coefficients between the strains and the loads can be obtained through the Equation (14).

(14) $$A=\frac{Cov(X,Y)}{\sqrt{Var\left[x\right]Var\left[y\right]}},$$

where *Cov*(*x*,*y*) is the covariance of *x* and *y*, *Var*[*x*] is the variance of *x*, *Var*[*y*] is the variance of *y* and *A* is the correlation coefficient between the loads and the strains. According to Equation (14), the correlation coefficient *A* can be calculated from Table 2. The correlation coefficients of each group samples (#1~#4) are 0.9296, 0.9251, 0.9457 and 0.9541, respectively. It is means there is a strong correlation between the loads and the detected strains.

## 4. Discussion

The goal of this paper is to achieve the measurement of the distribution of load-driven deformation and strain in the bone-implant interface. Aiming at this goal, a novel speckle interference imaging method for DSPI system was proposed, in which the samples are completely immersed into the PBS solution to realize the stability of liquid molecules on the macro probability. By using the method, it will reduce the molecular layer fluctuation on the detected surface, improve the speckle correlation.

The stability of the speckle is evaluated with the concepts of bio-speckles, co-occurrence matrix and moment of inertia. During the experiment process, the *IM* value is closely related to the experimental external factors, so the contrast experiments need to be carried out under the same conditions, such as light, temperature, etc.

Before the strain detection, the soft tissues will be removed, and the bone-implant interface will be exposed after removing soft tissues. Although the removal allows us to measure the strain distributions with DSPI, the limitation is that removing the soft tissues would affect the stress-strain relationship. In order to maintain the ex vivo mechanical conditions close to in vivo, the removal should be minimized.

Figure 8 shows the comparative fringe patterns which come from the bone interface responded to external loading under two different environmental conditions. These experimental results show that the liquid environment imaging method is feasible for bone interface strain detection. However, image noise comes from many factors, there are still lots of uncertain factors that affect image quality, such as the introduction of liquid environment may bring new noise.

Figure 9 shows the result of quantitative loading experiment which only deals with the out-of-plane strains. The detection of out-of-plane strains focusing on the bone-implant interface in response to external loading is major research issues in this paper. By properly selecting loading point, detecting section and the angle of the beam, the experimental results are more satisfactory. However, in-plane strain is also unavoidable during loading process. How to detect in-plane strain during loading will be the next problem to be studied.

The experimental data in Table 2 shows that there is a difference between the detected strains and the loads. The difference may be caused by the existence of non-rigid factors in the sample. From the experimental data of this paper, we can see that the bovine sample with a higher rigidity has a greater difference, while the pig sample with a lower rigidity has a smaller difference. The results indicate that the difference may be related to the rigidity of the material.

## 5. Conclusions

In this paper, we developed a new strain distribution sensing system for the bone-implant interface based on digital speckle pattern interferometry. In order to avoid speckle decorrelation caused by fresh bone samples with wet surfaces, a novel speckle interference imaging method was proposed in which the entire bone sample was immersed into a PBS solution to maintain the original physiological environmental, avoid dewetting process, and realize the stability of liquid molecules on the macro probability. With the proposed sensing system, high-resolution determinations of load-driven deformation patterns and strain distributions become possible. It is will be useful to evaluate computational simulations and improve selection of implant designs and materials. Furthermore, this new speckle interferometry imaging method proposed can improve speckle correlation. Thus, the proposed imaging method has general applications in biological research which involves the detection of load-driven deformation and strain on biomaterial surface in physiological environment.

## Figures and Tables

**Table 1:** The phase diagrams and strain diagrams.

Phase Diagrams	Three-Dimensional Reconstruction of Out-of-Plane Strain
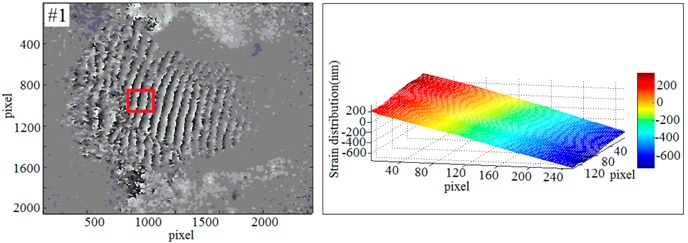	
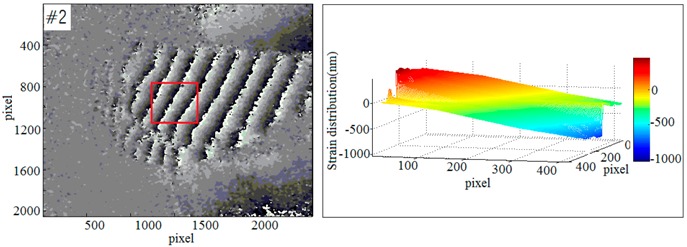	
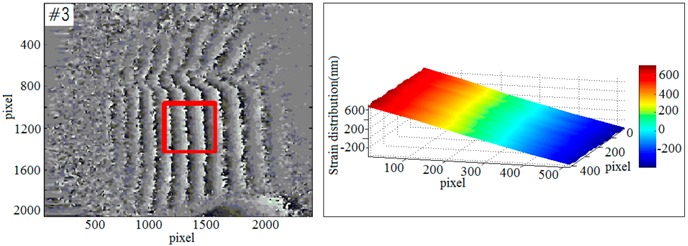	
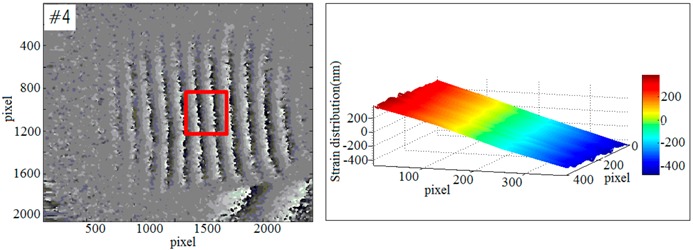	

**Table 2:** The repeated test data of samples (#1~#4).